# Antithrombotic and antiplatelet effects of plant-derived compounds: a great utility potential for primary, secondary, and tertiary care in the framework of 3P medicine

**DOI:** 10.1007/s13167-022-00293-2

**Published:** 2022-08-15

**Authors:** Peter Kubatka, Alena Mazurakova, Lenka Koklesova, Marek Samec, Juraj Sokol, Samson Mathews Samuel, Erik Kudela, Kamil Biringer, Ondrej Bugos, Martin Pec, Barbara Link, Marian Adamkov, Karel Smejkal, Dietrich Büsselberg, Olga Golubnitschaja

**Affiliations:** 1 Department of Medical Biology, Jessenius Faculty of Medicine, Comenius University in Bratislava, 03601 Martin, Slovakia; 2 Clinic of Obstetrics and Gynecology, Jessenius Faculty of Medicine, Comenius University in Bratislava, 03601 Martin, Slovakia; 3 Department of Pathological Physiology, Jessenius Faculty of Medicine, Comenius University in Bratislava, 03601 Martin, Slovakia; 4 Department of Hematology and Transfusion Medicine, Jessenius Faculty of Medicine in Martin, Comenius University in Bratislava, 03601 Martin, Slovakia; 5 Department of Physiology and Biophysics, Weill Cornell Medicine-Qatar, Qatar Foundation, Education City, 24144 Doha, Qatar; 6 Lambda Life JSC, 85101 Bratislava, Slovakia; 7 Department of Histology and Embryology, Jessenius Faculty of Medicine, Comenius University in Bratislava, 03601 Martin, Slovakia; 8 Department of Radiation Oncology, University Hospital Bonn, Rheinische Friedrich-Wilhelms-Universität Bonn, 53127 Bonn, Germany; 9 Department of Natural Drugs, Faculty of Pharmacy, Masaryk University, 61200 Brno, Czech Republic; 10 Predictive, Preventive and Personalised (3P) Medicine, Department of Radiation Oncology, University Hospital Bonn, Rheinische Friedrich-Wilhelms-Universität Bonn, 53127 Bonn, Germany

**Keywords:** Predictive preventive personalized medicine, Thrombosis, Antiplatelet effects, Anticoagulation, Circulation, Vascular disease, Ischemic stroke, Treated cancers, COVID-19, Comorbidities, Individualized patient profile, Primary, secondary, tertiary care, Natural drugs, Phytochemicals, Molecular pathways, Targets, Therapeutic modalities, Health policy

## Abstract

Thromboembolism is the third leading vascular disease, with a high annual incidence of 1 to 2 cases per 1000 individuals within the general population. The broader term venous thromboembolism generally refers to deep vein thrombosis, pulmonary embolism, and/or a combination of both. Therefore, thromboembolism can affect both – the central and peripheral veins. Arterial thromboembolism causes systemic ischemia by disturbing blood flow and oxygen supply to organs, tissues, and cells causing, therefore, apoptosis and/or necrosis in the affected tissues. Currently applied antithrombotic drugs used, e.g. to protect affected individuals against ischemic stroke, demonstrate significant limitations. For example, platelet inhibitors possess only moderate efficacy. On the other hand, thrombolytics and anticoagulants significantly increase hemorrhage. Contextually, new approaches are extensively under consideration to develop next-generation antithrombotics with improved efficacy and more personalized and targeted application. To this end, phytochemicals show potent antithrombotic efficacy demonstrated in numerous in vitro, ex vivo, and in vivo models as well as in clinical evaluations conducted on healthy individuals and persons at high risk of thrombotic events, such as pregnant women (primary care), cancer, and COVID-19-affected patients (secondary and tertiary care). Here, we hypothesized that specific antithrombotic and antiplatelet effects of plant-derived compounds might be of great clinical utility in primary, secondary, and tertiary care. To increase the efficacy, precise patient stratification based on predictive diagnostics is essential for targeted protection and treatments tailored to the person in the framework of 3P medicine. Contextually, this paper aims at critical review toward the involvement of specific classes of phytochemicals in antiplatelet and anticoagulation adapted to clinical needs. The paper exemplifies selected plant-derived drugs, plant extracts, and whole plant foods/herbs demonstrating their specific antithrombotic, antiplatelet, and fibrinolytic activities relevant for primary, secondary, and tertiary care. One of the examples considered is antithrombotic and antiplatelet protection specifically relevant for COVID-19-affected patient groups.

## Preamble

### Patho/physiology of hemostasis

Hemostasis is a complex process playing a fundamental role in preventing blood loss. It includes the close interplay of the vascular endothelium, platelets, and plasma coagulation factors. The interactions between platelets and components of the injured vascular wall play a crucial role in the activation/regulation of platelets. Platelets that form the hemostatic plug serve as a platform for the consequent events triggered by the coagulation factors that finalize the process of hemostasis [1]. In several cases, these normally physiologic processes can lead to pathological clot formation in the arteries or veins that are manifested as venous and arterial thromboembolism [2].

### Thromboembolism is one of the world’s leading vascular diseases

Thromboembolism is the third leading vascular disease, with a high annual incidence of 1 to 2 cases per 1000 individuals within the general population [3]. The broader term venous thromboembolism generally refers to deep vein thrombosis (DVT), pulmonary embolism (PE), and/or a combination of both (DVT/PE). In addition, thromboembolism can also affect the health status of other veins of the body, both central and peripheral ones. Less common sites of venous thromboembolism involve the arms, liver, brain, and kidneys. Arterial thromboembolism causes systemic ischemia by disturbing blood flow and oxygen supply to organs, tissues, and cells causing, therefore, apoptosis and/or necrosis in the affected tissues.

### Where does thromboembolism occur?

Arterial thromboembolism often occurs in the extremities (legs and feet) as well as in the life important organs: in the heart, causing a sudden heart attack (myocardial infarction, MI), and in the brain, causing ischemic stroke. In contrast, kidneys, intestines, and eyes are organs with a lower incidence of arterial thromboembolism observed [2, 4]. Thromboembolic diseases are associated with higher morbidity and mortality [5, 6], high rates of hospital readmissions [7], low health-related quality of life [8], and have a notable negative economic impact on society. In the pathologies mentioned earlier, antithrombotic drugs, which include antiplatelet therapies and anticoagulants, are standardly used in individuals. The pathophysiology of arterial thrombosis differs from that of venous thrombosis, as reflected by the different ways they are treated.

### Thrombosis treatments and adverse effects of antiplatelet drug administration

In broad terms, arterial thrombosis is treated with drugs that target platelets (such as cyclooxygenase inhibitor – acetylsalicylic acid; adenosine-diphosphate receptor antagonists – clopidogrel, ticlopidine, prasugrel, and ticagrelor; phosphodiesterase inhibitors – dipyridamole, cilostazol; and glycoprotein IIb/IIIa inhibitors – abciximab, tirofiban), and venous thrombosis is treated with drugs that target proteins of the coagulation cascade (such as vitamin K antagonist, low molecular weight heparins, unfractionated heparin, direct oral anticoagulants (DOACs), indirect parenteral factor Xa inhibitors) [9]. However, these drugs also demonstrate undesirable side effects. The major adverse effect of antiplatelet drug administration is an increased risk of bleeding complications. The gastrointestinal tract is one of the most common sites of bleeding. In addition, all types of anticoagulant treatment increase the risk of bleeding. Warfarin causes fetal loss and skin necrosis. Unfractionated heparins can cause osteoporosis, thrombocytopenia with or without thrombosis, and other rare reactions. Low molecular weight heparins are less likely to do so. Bleeding episodes constitute the main adverse effects of the DOACs [10, 11].

### Antithrombotic, antiplatelet, and fibrinolytic effects of phytochemicals

Comprehensive preclinical research demonstrates that specific phytochemicals and plant-derived extracts show significant antithrombotic, antiplatelet, and fibrinolytic activities [12]. These include flavonoids, alkaloids, saponins, coumarins, polyphenols, furan derivatives, iridoid glycosides, sesquiterpenes, and aporphines [13]. Flavonoids, as the most significant group of the above-mentioned phytochemicals, are known for their venotonic activity, but their mechanism of action remains incomplete. Supposedly, their activity is mediated by the regulation of prostaglandin metabolism. Specific phytochemicals may interfere with the arachidonic acid (AA) cascade and its metabolites, which are directly related to platelet aggregation regulation [14]. They also revealed significant antiplatelet activity via the modulation of a wide signaling network associated with the platelet activation blockage and calcium ionophore effects and increased fibrinolysis [15]. These data point to specific classes of phytochemicals as compounds that can reduce platelet functions, resulting in antithrombotic, antiplatelet, and fibrinolytic profiles and may play a clinically essential role in the prevention and therapy of cardiovascular diseases. Integrating relevant data in this topic associated with complex medical informatics and personalized medicine is highly recommended in preventing and treating thromboembolic events. Such medicinal approaches fall into the progressive concept of advanced health care tailored to the person [16]. Such a medical approach provides an advanced clinical strategy to improve individual outcomes and cost-efficacy in managing various pathologies [17–19].

## Working hypothesis and study aims in the framework of 3P medicine

Here, we hypothesized that specific antithrombotic and antiplatelet effects of plant-derived compounds might be of great clinical utility in primary, secondary, and tertiary care. To increase the efficacy, precise patient stratification based on predictive diagnostics is essential for targeted protection and treatments tailored to the person in the framework of 3P medicine.

Contextually, this paper aimed at critical review toward the involvement of specific classes of phytochemicals in antiplatelet and anticoagulation adapted to clinical needs. The paper exemplified selected plant-derived drugs, plant extracts, and whole plant foods/herbs demonstrating their specific antithrombotic, antiplatelet, and fibrinolytic activities relevant for primary, secondary, and tertiary care. One of the examples considered is antithrombotic and antiplatelet protection specifically relevant for COVID-19-affected patient groups.

## Source of the data

Data were obtained from the English-language biomedical literature by the use of “alkaloids,” “antiplatelet effects,” “anticoagulation,” “aporphine compounds,” “coumarins,” “COVID-19 patients,” “fibrinolytic,” “flavonoids,” “furan derivatives,” “iridoid glycosides,” “phytochemicals,” “plant extracts,” “plant foods,” “polyphenols,” “prevention,” “saponins,” “sesquiterpenes,” “therapy,” and “thrombosis,” or other associated terms as either a keyword or medical subject heading (MeSH) term in searches of the PubMed bibliographic database. In the special part, focusing on the antithrombotic and antiplatelet effects of phytochemicals, we emphasize the analysis of the most recent scientific papers from the years 2018–2022.

## Signal transduction pathways associated with platelet activation and platelet inhibition: possible targets for phytochemicals

**Fig. 1:**
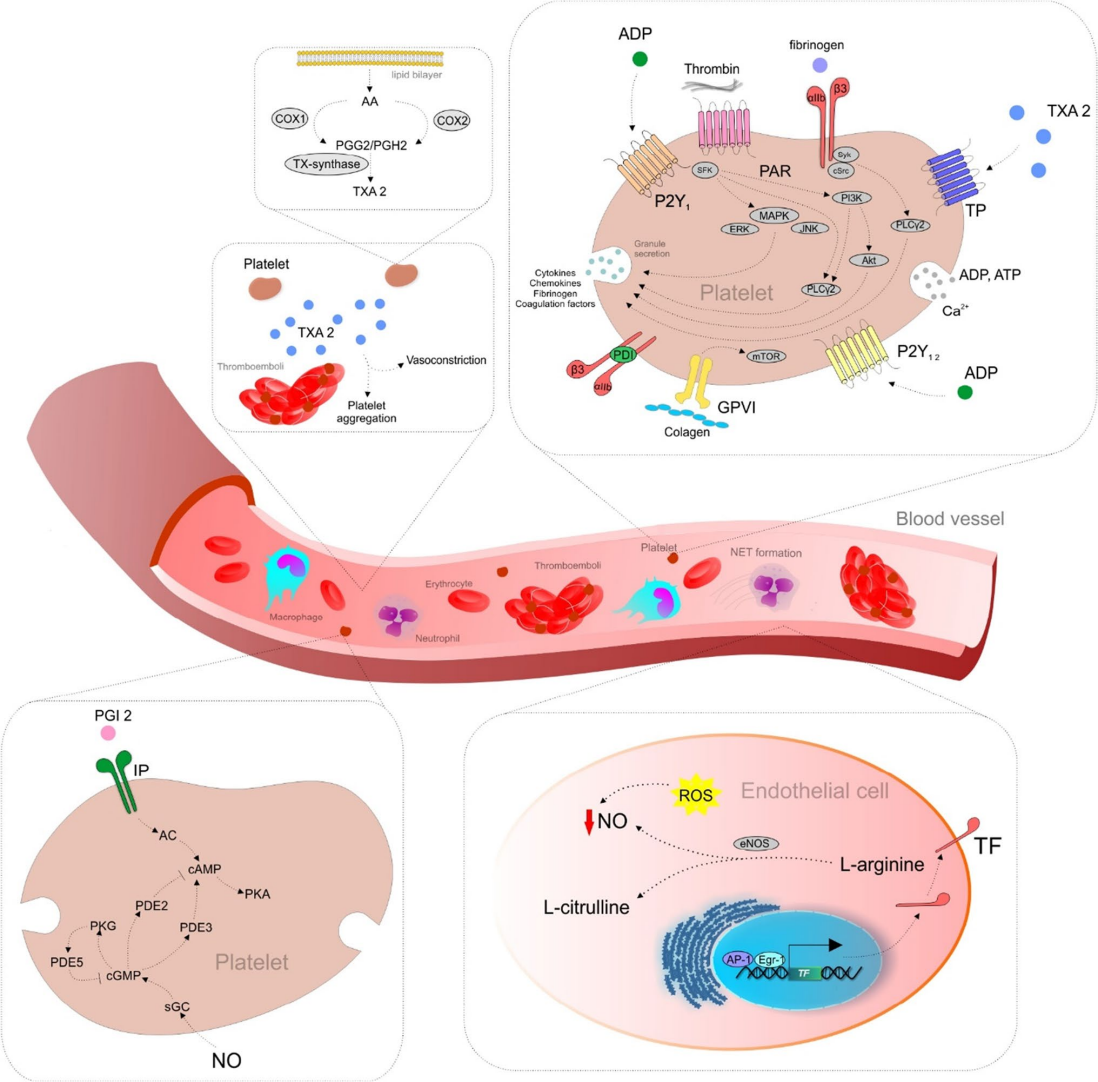
Signaling pathways associated with platelet activation. AA, arachidonic acid; COX1/2, cyclooxygenase 1/2; PGG2, prostaglandin G2; PGH2, prostaglandin H2; TX-synthase, thromboxane synthase; TXA2, thromboxane A2; ADP, adenosine diphosphate; PAR, protease-activated receptors; TP, thromboxane receptor; ATP, adenosine triphosphate; GPVI, glycoprotein VI; PDI, protein disulfide isomerase; SFK, Src family kinase; MAPK, mitogen-activated protein kinases; ERK; extracellular signal-regulated kinase; JNK, c-Jun N-terminal kinase; PLCγ2, phospholipase C gamma 2; PI3K, phosphoinositide 3-kinases; Akt, protein kinase B; mTOR, mammalian target of rapamycin; SYK, spleen tyrosine kinase; PGI2, prostaglandin I2; IP, prostacyclin receptor; NO, nitric oxide; AC, adenylyl cyclase; cAMP, cyclic adenosine monophosphate; PKA, cAMP-dependent protein kinase; sGC, soluble guanylyl cyclase; PKG, cGMP-dependent protein kinase; cGMP, cyclic guanosine monophosphate; PDE 2/3/5, phosphodiesterase 2/3/5; ROS, reactive oxygen species; eNOS, endothelial NOS; TF, tissue factor; AP-1, activator protein 1; Egr-1; early growth response 1

Studies describe the modulatory effects of specific phytochemicals on platelets mostly via signaling pathways affecting TXA2 release, platelet aggregation, or granule secretion [20]. Specific agonists targeting the key platelet receptors induce these activities [21]. Platelet activation is a crucial step in the pathogenesis of thrombosis. Besides the capacity of acetylsalicylic acid to inhibit the synthesis of TXA2 (an inducer of platelet aggregation and vasoconstrictor), it plays a crucial role in the treatment of thromboembolic disease and myocardial infarction [22]. AA derivatives (prostanoids and isoprostanes) are essential in the modulation of contractile and proliferative responses of vascular smooth muscle cells (VSMCs) and the aggregation of blood platelets. The platelet prostanoids normally functioning in vascular injury are also implicated in the progression of physiological hemostatic response to thrombotic occlusion. Indeed, AA is the precursor of TXA2 [23]. Figure 1 summarizes the specific signaling pathways associated with the activation of platelets and thrombotic processes as possible molecular targets for phytochemicals. In general, the role of signaling pathways in platelets is less well described compared to nucleated cells in the human organism, e.g., NFκB (nuclear factor kappa-enhancer of activated B cells), mTOR (mammalian target of rapamycin), and JAK (Janus kinase) [21].

Mentioned signaling pathways include G-protein coupled receptors for TXA2, ADP, and thrombin that activate platelets through protein kinase C (PKC) isoforms and phospholipase Cβ. Integrin αIIbβ3 mediates inside-out and outside-in signaling events, and cAMP signaling modulates thrombus formation [24]. Integrin αIIbβ3 activation via the protein kinase B (PKB or Akt), glycogen synthase kinase (GSK), and phosphoinositide 3-kinases (PI3K) is initiated via the P2Y1 and P2Y 12 ADP receptors. P2Y12 receptor activation is a crucial event in PI3K phosphorylation. On the other hand, P2Y1 receptor activation appears to induce high cytosolic Ca^2+^ levels conducive to increased activity of PI3K [25, 26]. The cyclooxygenase-induced synthesis of TXA2 depends on increased cytosolic Ca^2+^ levels linked with activating mitogen-activated protein kinases (MAPKs) isoforms, i.e., ERK and p38. Except for increased cAMP levels, Fuentes et al. described the antiplatelet activity caused by the PPARs agonist [26].

In addition, MAPKs, including ERK2, p38, and JNK1/2, are present and activated in platelets. In human platelets, JNK1 isoform is activated by thrombin, von Willebrand factor, and collagen, while αIIbβ3 can downregulate JNK1 activation by thrombin. Above all, JNK isoforms (JNK1/2/3) are associated with platelet adhesion and thrombus formation [27].

The phosphorylation of tyrosine-kinase-linked receptors, particularly the glycoprotein VI (GPVI) receptor for collagen, represents another way for platelet activation. Current research shows that mTOR plays an essential role in GPVI-dependent platelet activation and thrombus formation [28]. In addition, activation of αIIbβ3 outside-in signaling induces the phosphorylation of c-Src and spleen tyrosine kinase (Syk), and consequently, through PLCγ and PKC isoforms regulate platelet activation, granule secretion, platelet spreading, and clot retraction [29].

In addition, processes of thrombus formation can be modulated by the oxidation status of labile disulfide bonds in hemostatic processes. Disulfide bond formation is catalyzed by an oxidoreductase protein disulfide isomerase (PDI) or other thiol isomerases family members. PDI is also implicated in the regulation of αIIbβ3 activity [30]. Therefore, PDI, secreted by most of the cell types involved in thrombosis, plays a vital role in thrombus formation [31, 32].

The platelet inhibition in physiological conditions is associated with elevated cAMP- and cGMP-dependent protein kinases (PKA and PKG). Both kinases are activated by endothelial-derived prostaglandin I 2 (PGI_2_/IP receptor) and nitric oxide (NO), respectively. PGI_2_ and NO actions are mediated by platelet adenylyl and guanylyl cyclases, which synthesize cAMP and cGMP, respectively. Furthermore, deregulations in cAMP/cGMP signaling might contribute to platelet hyperreactivity. Finally, cAMP and cGMP are degraded by isoforms of phosphodiesterase (PDEs) that are included in these inhibitory processes and signaling restrictions within specific subcellular compartments [33].

In addition, NO released by the endothelium prevents platelet adhesion to the vessel wall, and when released by platelets, NO inhibits the recruitment of platelets to a growing thrombus [34]. Reactive oxygen species (ROS) are associated with decreased NO bioavailability, and oxidative stress contributes to endothelial dysfunction, progressive atherogenesis, and thrombosis [35]. The formation of the thrombus is associated with ROS through its effects on fibrinolysis, coagulation, proteolysis, and regulation of effector cells (platelets, endothelial cells, erythrocytes, mast cells, neutrophils, monocytes, or fibroblasts) [36].

Moreover, the initiator of the extrinsic coagulation cascade, tissue factor (TF) is considered the primary cause of atherothrombosis, atherosclerotic plaque rupture, and subsequent thrombosis. TF is regulated mainly at the transcriptional level, while binding sites for transcriptional factors, including activator protein-1 (AP-1) and early growth response-1 (Egr-1), are localized in human TF promoters [37].

## Phytochemicals with protective effects against thrombosis

Phytochemicals are widely distributed in vegetables, fruits, legumes, whole grains, seeds, and herbs. Examples of phytochemicals include phenolics, alkaloids, sulfur-containing substances, and terpenoids [38]. Flavonoids and non-flavonoid compounds, including phenolic acids, coumarins, stilbenes, and lignans, represent the main subgroups of phenolics [39–41].

### Flavonoids

Flavonoids represent a large group of phenolic compounds [42] that are widely found in fruits, vegetables, berry-based beverages, and many medicinal plants. The classification of flavonoids is associated with the chemical structure, oxidation level, and pattern of substitution of ring C (heterocyclic pyrane), while the substitution of rings A and B (benzene) defines the individual compounds within the subclasses. Flavonoids are therefore classified as flavanols, flavonols, flavones, flavanones, isoflavonoids, anthocyanins, and chalcones [43]. Flavonoids are clinically associated with numerous biological activities, including anti-inflammatory, antioxidant, and potential anti-cancer effects [42, 44–46]. Moreover, flavonoids are discussed as potentially effective antithrombotic agents [47, 48].

Quercetin is one of the most widely distributed and studied flavonoids belonging to flavonols. It is found in large quantities in apples, onions, or red wine [43]. Quercetin exists primarily in glycosylated forms such as quercitrin [49]. Quercetin and its derivatives (isorhamnetin and tamarixetin) exert potent antithrombotic activity and effects on platelet inhibition demonstrated through the suppression of platelet aggregation and the modulation of early activatory processes in vitro, including the inhibition of granule secretion, cytosolic calcium elevation, and early signaling events downstream of GPVI, as well as the modulation of integrin αIIbβ3 function. Moreover, quercetin-derived flavonols enhanced the antiplatelet effects of acetylsalicylic acid in vitro and inhibited thrombus formation in vivo [50]. Similarly, quercitrin inhibited platelet aggregation, granule secretion, ROS generation, and intracellular calcium mobilization in arterial thrombosis models.

Furthermore, quercitrin suppressed outside-in signaling of αIIbβ3 integrin and inhibited thrombus formation in vivo and in vitro (on collagen-coated surfaces under arteriolar shear). Indeed, the inhibitory effects were mediated by the inhibition of GPVI-modulated platelet signal transduction during cell activation, thus regulating platelet activation [49]. In addition, using quercetin 3,7,3ʹ,4ʹ-tetrasulphate, extracted from leaves of *Flaveria bidentis* (L.) Kuntze, showed antithrombotic effects in vivo from high (100 mg/kg/i.p) to low (25 mg/kg/i.p) concentrations in a dose-dependent manner, demonstrated through prolonged bleeding time and increased blood loss in a model of pulmonary thromboembolism [51]. Also, the flavonol kaempferol showed potent antithrombotic effects and antiplatelet activation in vitro, in vivo, and ex vivo. The observed effects of kaempferol included decreased enzymatic activity of coagulant factors in the coagulation cascade, specifically thrombin and activated factor X (FXa), and fibrin clot formation, platelet activation, prolonged activated partial thromboplastin time (APTT), and survival of thrombotic challenges. Indeed, the effects on APTT and prothrombin time (PT) reflected the anticoagulant activity of this phytosubstance [52]. Rutin, isolated from *Dendropanax morbifera* Leville, exerted potent antithrombotic effects in vitro and in vivo through inhibition of thrombin activity, fibrin clotting, prolongation of APTT and PT prolongation, and protection from thrombotic challenge [53]. Current research highlights the popularity of nanomedicines to load and deliver the anticoagulants directly to the target, reduce the dose of anticoagulant agents and improve antithrombic efficacy, and decrease the complications represented mainly by hemorrhage, or improve the stability and aqueous solubility of the agent. Wu and colleagues evaluated the antithrombic effects of rutin-loaded silver nanoparticles (Rutin@AgNPs). The authors described the potent anticoagulant activity of Rutin@AgNPs, demonstrated through prolonged APTT and PT, as well as the inhibition of thrombosis in a carrageenan-induced venous thrombosis mouse model. Effects were associated with the described capacity of rutin to target and inhibit PDI, thus resulting in the blockage of platelet accumulation and fibrin generation, accompanied by a reduction of inflammation induced by carrageenan [31].

The main representatives of flavanones, a class of flavonoids mainly found in citrus fruits, are, among others, hesperidin and naringin [43, 54]. Ikemura et al. (2012) evaluated the effects of hesperidin, glucosyl hesperidin (G-hesperidin) – a water well-soluble derivative of hesperidin – and naringin on blood pressure and cerebral thrombosis in stroke-prone spontaneously hypertensive rats. The authors found a decreased thrombotic tendency in cerebral blood vessels, effects on oxidative stress demonstrated through reduced marker of oxidative stress 8-hydroxy-2ʹ-deoxyguanosine (8-OHdG), increased production of NO metabolites, and increased vascular relaxation in a stroke-prone spontaneously hypertensive rats. Potent antioxidant capacity protects endothelial function from ROS [35]. Moreover, a recent study by Haggag et al. suggested the potential effects of hesperidin against venous thromboembolism in association with COVID-19 [55].

Guerrero et al. (2005 and 2007) reviewed that flavonoids could inhibit platelet function by binding to the TXA2 receptor [56, 57]. The flavone apigenin is one of the most common flavonoids widely found in fruits and vegetables [43]. Apigenin exerted a potent capacity to modulate platelet reactivity. Apigenin inhibited platelet adhesion and thrombus formation and synergized with acetylsalicylic acid in suppressing the AA pathway in vitro. The inhibitory effects of apigenin could at least rely on TXA2 receptor antagonism. These results support the potential combined use of acetylsalicylic acid and flavonoids in patients who suppress TXA2 failed.

Similar to apigenin, isoflavone genistein and flavan-3-ol catechin also diminished thrombus formation in the same model [22]. Furthermore, wogonin, a flavone isolated from *Scutellaria baicalensis* Georgi, exerted a potent capacity to target the initiator of extrinsic coagulation cascade – TF. Wogonin inhibited ERK/Egr-1- and JNK/AP-1-mediated transactivation of TF promoter activity, resulting in the downregulation of TF expression and activity induced by inflammatory mediators in human endothelial cells [37]. Similarly, wogonin and its glycoside wogonoside showed antithrombotic effects in vitro and in vivo, demonstrated through anticoagulant activity (prolonged APTT and PT), inhibited fibrin polymerization, mouse platelet aggregation induced by thrombin, thrombin activity and production, FXa activity, and prolongation of tail bleeding time in mice [58].

The main phytochemicals in green tea are known as green tea catechins (GTC). A flavanol derivative (-)-epigallocatechin gallate (EGCG) represents the main GTC. Antithrombotic activity of GTC was described at the end of the twentieth century by Kang et al. (1999), who demonstrated the effects of GTC and EGCG in the protection from paralysis or death by pulmonary thrombosis and the prolongation of the mouse tail bleeding time of conscious mice in vivo and inhibition of platelet aggregation in vitro and ex vivo [59]. Also, epicatechin inhibited the maximal platelet aggregation induced by adenosine diphosphate, thrombin receptor activating peptide, epinephrine, and collagen, reduced endogenous thrombin potential, and improved fibrinolysis in analyses evaluating plasma samples from healthy volunteers [15]. Cocoa is a rich source of bioactive compounds, including flavan-3-ols such as epicatechin and catechin. Montagnana et al. (2018) studied healthy volunteers and demonstrated that dark chocolate modulates platelet function via flavan-3-ol metabolites. Due to the crucial role of platelets in arterial and venous thromboembolism, the authors suggested the beneficial part of dark chocolate consumption in subjects with an increased risk of thrombosis [60]. In addition, a study conducted on healthy men demonstrated flavonoid-rich dark chocolate blunted acute prothrombotic response to psychosocial stress through attenuated stress reactivity of the hypercoagulability marker D-dimer [61], a marker for coagulation cascade activation and fibrinolysis [62]. Besides, elevations of plasma D-dimer mark an increased risk of thrombosis, especially in cancer patients [63]. Flavonoids show potent antithrombotic capacity also in cancer patients. Isoquercetin exerted an ability to target extracellular PDI and improve coagulation markers in advanced cancer patients [63].

### Phenolic acids and furan derivatives

Phenolic acids are one of the main classes of plant phenolic compounds characterized by an aromatic skeleton and carboxylic group [64]. Phenolic acids are divided, depending on their structure, into hydroxybenzoic and hydroxycinnamic acids [65].

Caffeic acid is found in different plant species and represents an essential component of coffee, tea, or wine. Caffeic acid exerts numerous beneficial effects on human health, mediated by its antioxidant and anti-inflammatory activities, which were documented in many preclinical and clinical studies [66–70]. Moreover, caffeic acid may affect pathological cascades associated with thrombosis [71]. Antithrombotic activities of caffeic acid were observed via inhibition of platelet aggregation. Caffeic acid significantly reduced thrombin-induced platelet aggregation, Ca^2+^ mobilization, and P-selectin expression—furthermore, caffeic acid regulated the ability of integrin αIIbβ3 to bind fibrinogen and accelerated cAMP generation. In addition, caffeic acid suppressed thrombin-induced Akt and ERK phosphorylation in platelets [72]. Similarly, Lee et al. identified antiplatelet activity of caffeic acid mediated by inhibition of Ca^2+^ mobilization via inositol 1,4,5-trisphosphate (IP_3_R) phosphorylation and decreasing TXA_2_ production via COX-1 inhibition in washed platelets from rats [73]. Curcumin (diferuoylmethane), a yellow pigment extracted from *Curcuma longa* L., possesses numerous beneficial properties for human health [74]. Anticoagulant activities of curcumin and its derivate bisdemethoxycurcumin (BDMC) were documented to inhibit thrombin and FXa generation in the HUVEC cell line. Moreover, curcumin and BDMC significantly prolonged clotting time in plasma-based coagulation APTT and PT tests [75].

In large quantities obtained from cinnamon, cinnamic acid is characterized by low toxicity and a broad range of biological activities associated with health benefits. It represents an essential component of safflower injection (known in traditional Chinese medicine). Anticoagulant features of cinnamic acid were documented in vitro by measuring APTT and PT. Three active ingredients of safflower injection (*p*-hydroxybenzaldehyde; (8*Z*)-decaene-4,6-diyne-1-*O*-D-glucopyranoside; and *p*-hydroxycinnamic acid) were evaluated according to their APTT against human plasma. Among analyzed components, *p*-hydroxycinnamic acid showed the most significant prolonging of APTT tested on human plasma [76]. Ferulic acid, widely distributed in numerous plants, demonstrated antithrombotic activities both in vitro and in vivo. Choi et al. confirmed the antithrombotic role of ferulic acid mediated by prolonged recalcification time in plasma coagulation. In vivo analysis identified the role of ferulic acid in downregulating αIIbβ3/fibrinogen complex (FIB) expression as well as in phosphorylating Akt in thrombin-stimulated platelet activation [77]. Similarly, ferulic acid exerted an antithrombotic effect in vivo and in vitro via different mechanisms of action. Oral administration of ferulic acid reduced the risk of death due to pulmonary thrombosis and delayed clotting time in mice. In addition, ferulic acid dose-dependently suppressed platelet aggregation in vivo and in vitro and affected levels of thromboxane B2 (TXB2), cAMP, cGMP, and phosphorylation of MAPKs and PDE. Interestingly, this phenolic acid influenced Ca^2+^ mobilization in washed rat platelets, which may subsequently affect the activation of platelet integrins, secretion of granules, or platelet shape changes [78, 79]. Moreover, ferulic acid significantly prolonged whole blood coagulation time (WBCT) in vitro [80]*.*

Furan is a 5-membered heterocyclic, oxygen-containing, unsaturated ring compound [81]. Furans, benzofurans, and their reduced forms represent common structural motifs in naturally occurring compounds [82]. A natural breakdown product of glucose and fructose-containing foods (fruit juices), 5-(hydroxymethyl)furfural, reduced the effects of hypoxia on sickle cell trait that is commonly associated with increased risk for venous thromboembolism and chronic kidney disease; 5-(hydroxymethyl)furfural also decreased blood rheology in vitro and restored near-normal flow velocities at very low oxygen [83]. Moreover, Dan Zhi tablets, commonly used in traditional Chinese medicine, exert antiplatelet activity that could lead to cerebral ischemic injury protection. Dan Zhi tablets contain various naturally occurring compounds, including furan sulfonic acids, phenolic acids, tanshinones, flavonoids, saponins, and phthalides. Dan Zhi tablet inhibited in vitro prostaglandin G/H synthase 1 (PTGS1) activity and platelet aggregation. Moreover, the Dan Zhi tablet reduced ex vivo platelet aggregation and reduced thromboxane A2 (TXA2) level in a rat model of middle cerebral artery occlusion. Dan Zhi tablets’ other beneficial properties prevented thrombus formation in an acute pulmonary thromboembolism mice model [84].

### Stilbenes

Stilbenes are naturally occurring non-flavonoid phenolics characterized by the presence of 1,2-diphenylethylene nuclei [85]. This group of phenolic compounds exerts a multifunctional impact on human health via modulation of antioxidant, anti-inflammatory, and cardioprotective cascade [86]. Resveratrol (3,4ʹ,5-trihydroxy-*trans*-stilbene) represents the most extensively studied stilbene which possesses a wide range of health-associated impacts, including antithrombotic and antiplatelet effects [87]. Resveratrol exerted a synergic effect combined with warfarin (routinely used to prevent blood from clotting) in rats. Orally administrated resveratrol improved pharmacokinetics and anticoagulant activity of warfarin via suppression of breast cancer resistance protein (BCRP) and cytochrome P450 family 2 subfamily C member 9 (CYP2C9) [88].

Similarly, resveratrol enhanced the anticoagulant capacity of warfarin in animal models using male C57BL/6 J mice [89]. Furthermore, resveratrol showed anticoagulated, anti-inflammatory, and antifibrinolytic roles analyzed in vitro using the HUVEC cell line. In-depth analysis revealed that resveratrol decreased interleukin 8 (IL-8), tissue plasminogen activator-1 (t-PA-1), and von Willebrand factor expression and secretion, as well as inhibited activity of factor VIII [90]. Additionally, based on its potent antithrombotic effect, a study by Xu et al. suggested the role of resveratrol in reducing the incidence of portal vein system thrombosis (PVST) after splenectomy in an animal model of fibrosis via decreasing platelet aggregation, generation of ROS in platelets, and increasing NO synthesis and platelet apoptosis [91].

### Coumarins

Coumarins are naturally occurring α-benzopyrone derivatives [92]. The antithrombotic effects of many coumarins, including daphnetin [93], semisynthetic warfarin, and other 4-hydroxycoumarins [94], such as phenprocoumon [95], were described and utilized in clinical practice. Recent studies focus on the more specific effects of coumarins and their mechanisms of action. Esculetin, a bioactive 6,7-dihydroxy derivative of coumarin, prevented thrombosis by inhibiting PLCγ2-PKC-AKT activation in human platelets. Moreover, esculetin reduced collagen- and arachidonic acid-induced platelet aggregation, ATP release, P-selectin expression, and hydroxyl radical formation. In a mouse model, esculetin reduced mortality related to acute pulmonary thromboembolism and increased the occlusion time in thrombotic platelet plug formation [96]; 3-(5-hydroxy-2,2-dimethyl-chroman-6-yl)-*N*-{2-[3-(5-hydroxy-2,2-dimethyl-chroman-6-yl)-propionylamino]-ethyl}-propionamide (C3), a newly synthetized coumarin derivative, prevented pulmonary thromboembolism and death in mice. In a model of the arteriovenous shunt, reduced thrombus weight was also observed. Moreover, in platelet-rich rat plasma, C3 reduced platelet aggregation, and in a hamster model of chronic dyslipidemia, the C3 administration reduced whole-blood aggregation [97].

### Alkaloids

Alkaloids are naturally occurring nitrogen-containing plant metabolites [98]. Alkaloids are characterized by a great structural diversity that can be divided into 5 groups: true alkaloids, protoalkaloids, polyamine alkaloids, peptide and cyclopeptide alkaloids, and pseudoalkaloids [99]. Some alkaloids showed antithrombotic and antiplatelet effects. Rutaecarpine, an alkaloid from *Tetradium ruticapum* (A.Juss.) T.G.Hartley (syn. *Evodia rutaecarpa*), prevented platelet activation in humans and reduced microvascular thrombosis in mice. Rutaecarpine exerted an antiplatelet activation effect by inhibiting PLCγ2/PKC and PI3K/Akt/GSK3β pathways. Moreover, rutaecarpine reduced P-selectin expression, ATP release, Ca^2+^ immobilization, and hydroxyl radical formation [100]. The consumption of coffee purine-like alkaloids such as caffeine reduced the risk of venous thrombosis (30% lower risk) compared with the no coffee consumption group. These results seem to be associated with lowering hemostatic factors, including the von Willebrand factor and FVIII, in coffee consumers [101].

Aporphine alkaloids are naturally occurring chemical compounds from the group of alkaloids derived from isoquinoline, which are widely distributed in the Annonaceae, Lauraceae, Magnoliaceae, and Menispermaceae [102]. Rhizoma of *Corydalis yanhusuo* W.T.Wang contains nearly 40 alkaloids, aporphine alkaloids among them. Five alkaloids extracted from *Corydalis rhizoma*, including aporphine glaucine, isochinolines dehydrocorydaline, canadine, tetrahydrocoptisine, and corydaline, inhibited in rabbit platelets thrombin-induced platelet aggregation in a low dose. In contrast, other alkaloids (protoberberine palmatine and tetrahydropalmatine) did not exert antiplatelet effects. Further investigations of these effects are needed [103].

### Saponins

The saponins are glycosides derived from triterpenes and steroids. Several plants from families such as Araliaceae, Fabaceae, Polygalaceae, Campanulaceae, Dioscoreaceae, Liliaceae, and Scrophulariaceae produce saponins as secondary metabolites [104].

Among others, a spirostane saponin from *Liriope muscari* (Decne.) L.H.Bailey, assigned as D39, demonstrated antithrombotic activity in vitro and in vivo by targeting non-muscular myosin heavy chain IIA (NMMHC IIA). D39 inhibited procoagulant activities and tissue factor expression in HUVECs. Furthermore, D39 decreased thrombus weight in inferior vena cava-ligated mice. Inhibition or knockdown of NMMHC IIA decreased tissue factor expression and deep vein thrombosis, primarily due to a modulation of the Akt/GSK3β-NF-κB signaling pathway [105].

Diosgenin, the saponin extracted from the rhizome of *Dioscorea zingiberensis* C.H. Wright, possesses antithrombotic activity. In vivo study revealed that its synthetic derivative disaccharide saponin diosgenyl-β-D-galactopyranosyl-(1 → 4)-β-D-glucopyranoside inhibited platelet aggregation and factor VIII activities and prolonged APTT in rats. This compound also increased the protection rate in mice, suggesting that steroidal saponins exerted antithrombotic activity [106].

Using a mixture of panaxatriol saponins extracted from *Panax notoginseng* (Burkill) F.H.Chen demonstrated antiplatelet activity in rabbit and human platelets. Panaxatriol saponins reduced rabbit platelet aggregation induced by different agonists such as collagen, thrombin, or ADP. The three main panaxatriol ginsenosides (Rg1, Re, and R1) revealed antiplatelet activity on rabbit platelet aggregation but without synergistic effects when combined. Similarly, the antiplatelet activity of the panaxatriol saponin mixture and its ginsenosides was observed on human platelets. Furthermore, pre-treatment with panaxatriol saponin mixture decreased the agonists-induced intracellular calcium mobilization by suppressing ERK2 and p38 phosphorylation [107].

### Iridoid glycosides

Based on the structure, the iridoids, a class of cyclopentane pyran monoterpenes, can be divided into four groups: iridoid glycosides, secoiridoid glycosides, non-glycosidic iridoids, and *bis*-iridoids [108]. Almost 600 iridoid glycosides are described from 57 families of plants [109]. Some iridoids showed promising antithrombotic activities.

*Gardenia jasminoides* J. Ellis with the main constituents iridoid glycosides and crocins prolonged bleeding time and inhibited platelet aggregation and thrombosis in rats [110]. Another study revealed that geniposide, one of the constituents of *G. jasminoides*, exerted antithrombotic activity in mice. Geniposide and its metabolite genipin prolonged the time required for thrombotic occlusion and inhibited platelet aggregation through the inhibition of phospholipase A(2) (PLA(2)) activity [111].

Iridoid glycosides extracted from Zhizi (*Gardeniae fructus*) showed antithrombotic action by inhibiting platelet aggregation and reducing arterial thrombus in rats. Moreover, iridoid glycoside prolonged the thrombin time but only at a higher dose [112].

### Sesquiterpenes

Sesquiterpenes are C15-terpenoids built from three isoprene units. The basic sesquiterpenic skeleton is often modified by oxidation to form lactones, alcohols, acids, aldehydes, and ketones [113].

Sesquiterpene glycoside 3-O-α-L-rhamnopyranosyl-(1 → 4)-α-L-rhamnopyranosyl-(1 → 2)-[α-L-(4-*trans*-feruloyl)-rhamnopyranosyl-(1 → 6)]-β-D-glucopyranosyl nerolidol and ferulic acid isolated from the leaves of *Eriobotrya japonica* (Lindley) inhibited tissue factor activity and elongated the prothrombin time in the presence of tissue factors in a dose-dependent manner [114].

Another sesquiterpene compound, curdione, isolated from the essential oil of *Curcuma aromatica* (Salisb), inhibited human platelet aggregation through downregulation of the phosphorylated AMPK (P-AMPK) and P-integrin and reduction of vinculin/talin-mediated integrin αIIbβ3 signaling pathway [115].

Nootkatone, a sesquiterpenoid commonly found in grapefruit, inhibited the prothrombotic effect induced by diesel exhaust particles in platelet aggregation in whole blood (in vitro) and pial arterioles and venules (in vivo). Furthermore, nootkatone inhibited the plasma concentration of fibrinogen, plasminogen activator inhibitor-1, IL-6, and lipid peroxidation, preventing the shortening of the activated partial thromboplastin time and prothrombin time. Finally, nootkatone mitigated thrombogenicity, oxidative stress, and DNA damage induced by diesel exhaust particles by activating nuclear factor erythroid-derived 2-like 2 and heme oxygenase-1 [116].

### Sulfated polysaccharides

Sulfated polysaccharides, including galactans, ulvans, fucans, and fucoidans, represent heterogeneous structures with known anticoagulant activity. Seaweeds biosynthesize sulfated polysaccharides as a key component of their cell walls [117].

Sulfated galactans from the red alga *Acanthophora muscoides* exerted serpin-independent anticoagulant activities and FXII-related procoagulant effects. Moreover, sulfated galactans reduced arterial thrombus formation; however, opposite effects were demonstrated on venous thrombosis [118]. Moreover, sulfated D-galactans in the red algae *Botryocladia occidentalis* exerted anticoagulant activity. They could prevent thrombosis at a lower dose (at doses up to approximately 0.5 mg/ kg body weight) through enhanced thrombin and factor Xa inhibition by heparin cofactor II or antithrombin. On the other hand, at a higher dose, sulfated D-galactans lost their antithrombotic and anticoagulant effects and acted as a potent inducer of platelet aggregation. In platelet-depleted animals, the antithrombotic effect of a higher dose of sulfated D-galactan was restored, leading to the inhibition of thrombus formation [119]. Furthermore, sulfated pyranosic (1- > 3)-β-L-arabinan obtained from *Codium vermilara* (Bryopsidales) revealed anticoagulant activity through the direct interaction with thrombin [120].

### Plant foods with potent antithrombotic effects

*Ginkgo biloba* L. is a traditional medicinal plant widely used for its health beneficiary effects for more than 2000 years. *Ginkgo biloba* is associated with numerous phytochemicals, including flavonoids, terpenoids, and alkylphenols [121]. Importantly, *Ginkgo biloba* is used to prevent and treat cardiovascular diseases and thrombosis. Indeed, Chen et al. (2019) recently evaluated the mechanisms beyond the antithrombotic efficacy of *Ginkgo biloba* constituents. The authors described the thrombin-inhibitory activity of Ginkgo constituents, especially biflavones (ginkgetin, isoginkgetin, bilobetin, and amentoflavone) and flavonoids (luteolin, apigenin, quercetin, kaempferol, and isorhamnetin). Moreover, the molecular docking method showed that these biflavones could occupy the active cavity with strong interactions of salt bridges and hydrogen bonds. As suggested by mass spectrometry-based lysine labeling reactivity assay, the biflavones could bind on human thrombin at exosite I rather than exosite II. Therefore, *Ginkgo biloba* biflavones act as potent inhibitors of human thrombin and could be used as novel thrombin inhibitors [122].

Furthermore, *Ginkgo biloba* Extract 50 (GBE50) in combination with aspirin resulted in enhanced antiplatelet effects (demonstrated through both synergistic and additive effects in restraining platelet aggregation) in vitro [123]. In addition, *Ginkgo biloba* extract showed antithrombotic effects on endothelial cells demonstrated through increased thrombomodulin expression and tissue-type plasminogen activator secretion in the HUVECs cell model. At the same time, Krüppel-like factor 2 (KLF2) is considered a vital factor in these mechanisms. Indeed, KLF2 is described to possess a beneficial role in promoting thrombomodulin expression to prevent thrombosis formation [124].

Chamomilla is a popular herbaceous plant native to Europe and Western Asia, and the plant is known for its potent medicinal effects. The main phytochemicals of the chamomilla include phenolic compounds (especially flavonoids quercetin, apigenin, or luteolin, among others) [125]. Chamomilla demonstrates potent effects on platelet inhibition. Pierre et al. (2005) evaluated the inhibitory effects of aqueous extract of herbs on human platelet aggregation. They showed that chamomilla aqueous extract possesses potent antiplatelet effects, for example, inhibition of ADP-induced and collagen-induced platelet aggregation in vitro [126]. In addition, polysaccharide-polyphenolic conjugates isolated from *Matricaria chamomilla* L. (MC) are also being investigated to act on blood platelets. The treatment of platelet-rich plasma from healthy donors with polyphenolic-polysaccharide conjugates from MC resulted in decreased platelet aggregation, and MC also reduced platelet aggregation in platelet-rich plasma from patients with cardiovascular disorders. Moreover, MC showed cytotoxicity effects on human blood platelets, mouse fibroblast cultures L929, and human lung cells A549. Therefore, MC compounds represent a potential source of the new antiplatelet agent [127].

*Allium* species are characterized by numerous bioactive compounds with significant antiproliferative, anti-inflammatory, and antioxidant activity documented in vitro and in vivo [128–131]. Additionally, the mixture of phytochemicals occurring in garlic could be potentially beneficial in the treatment and prevention of cardiovascular disease and thrombosis [132–134]. Antiplatelet activity of two *Allium* species (*A. ursinum* and *A. sativum*) extracts was evaluated in a study by Hiyasat and coworkers. Authors revealed the antiaggregatory role of both *Allium* species via inhibition of the ADP pathway in vitro [135]. In another study, Lorigooini et al. evaluated the antiplatelet aggregation effect of seven *Allium* species (*A. shelkovnikovii*, *A. jesdianum*, *A. haemanthoides*, *A. vavillovi*, *A. atroviolaceum*, *A. hirtifolium*, and *A. ampeloprasum*). Acquired data showed that *A. atroviolaceum* exerted a maximum antiplatelet aggregation effect compared to other allium species [136]. Moreover, the antiplatelet activity of garlic tablets was compared to the cardioprotective dose of aspirin in a randomized clinical trial conducted on healthy volunteers. Experimental data showed no significant effect of platelet aggregation after the consumption of garlic tablets in any quantity [137].

The mixture of phytochemicals occurring in *Silybum Marianum L*. has numerous beneficial effects on human health. Recent evidence identified its anticancer [138], anti-apoptotic, and anti-inflammatory efficacy [139]. Moreover, *Silybum Marianum L.* and its constituents (e.g., flavonolignans) affect platelet aggregation. Bijak et al. (2016) analyzed the inhibitory effect of major flavonolignans (silybin, silychristin, and silydianin) on ADP-induced platelet activation. All tested flavonolignans significantly and dose-dependent manner suppressed platelet activation [140]. Moreover, silybin, silychristin, and silydianin inhibited platelet aggregation, COX activity, and decreased levels of malondialdehyde and thromboxane A_2_ in vitro [141]. Similarly, flavonolignans and their sulfate conjugants impacted platelet aggregation and blood vessels in isolated rat aorta and human blood. Analyzed flavonolignans showed potent vasorelaxant effects ex vivo, but antiplatelet activity was relatively weak [142].

Table 1 summarizes preclinical and clinical evidence of the antithrombotic effects of phytochemicals.

**Table 1:** Antithrombotic effects of phytochemicals

Phytochemical		Study design	Effects/results			Ref
**Flavonoids**						
Quercetin and its metabolites (tamarixetin and isorhamenetin)		In vitro and in vivo (C57/BL6 mice) models of thrombus formation	↓ Platelet aggregation triggered by collagen, ADP-, U46619- (a stable TXA2 analog), and thrombin (GPCR-mediated pathway inhibition); ↓ activatory processes (granule secretion, integrin αIIbβ3 function, Ca^2+^ mobilization, and Syk/LAT phosphorylation downstream of GPVI); ↑ antiplatelet effects of acetylsalicylic acid			[50]
Quercitrin		In vitro, in vivo (wild-type C57BL/6 mice)	↓ Platelet aggregation and ATP secretion; ↓ CRP-induced αIIbβ3 integrin activation and P-selectin exposure; ↓ Ca^2+^ mobilization; ↓ CRP-induced intracellular ROS formation; ↓ platelet αIIbβ3 outside-in signaling; ↑ downregulation of GPVI signaling events; ↓ thrombus formation in vivo and in vitro (on collagen-coated surfaces under arteriolar shear)			[49]
Quercetin 3,7,3′,4′-tetrasulphate		In vivo model of pulmonary thromboembolism (C57BL/6 mice)	Antithrombotic effects; ↑ blood loss; ↑ bleeding time			[51]
Kaempferol		In vitro, ex vivo (ICR MICE), in vivo (male ICR mice, Sprague–Dawley rats)	↓ Enzymatic activities of thrombin and FXa; ↓ fibrin polymer formation; ↓ fibrin clot formation; ↑ APTT (anticoagulant activity); ↓ platelet activation; ↓ thrombin-induced ERK1/2, p38, JNK1/2, and AKT activation; ↑ survival of thrombotic challenge with collagen and epinephrine (or thrombin) in mice; ↓ vascular occlusion of the carotid artery in FeCl_3_-induced carotid arterial thrombus model in rats			[52]
Rutin		In vitro and in vivo (male ICR mice)	↓ Fibrin clotting; ↓ thrombin activity; ↓ FXa (slight decrease); ↓ blood clot; ↑ APTT and PT prolongation (anticoagulant activity); ↓ platelet activation; ↑ survival of thrombotic challenge with collagen and epinephrine (or thrombin) in mice			[53]
Rutin@AgNPs		In vitro and in vivo (ICR mice)	↑ APTT and PT (anticoagulant activity); ↓ thrombus formation in carrageenan-induced venous thrombosis mouse model (↓ PDI → blockage of platelet accumulation and fibrin generation; anti-inflammatory effects → ↓ inflammation caused by carrageenan)			[31]
Hesperidin, glucosyl hesperidin, and naringin		In vivo (stroke-prone spontaneously hypertensive rats)	↓ Thrombotic tendency; ↑ antioxidant effects (↓ 8-OHdG); ↑ NO metabolites; ↑ vascular relaxation			[35]
Apigenin		In vitro	↓ Platelet adhesion and thrombus formation; ↓ AA pathway in synergy with acetylsalicylic acid (TXA 2 receptors antagonism); ↓ thrombus formation (also by genistein and catechin)			[22]
Wogonin		In vitro (ECs)	↓ TF expression and activity (anticoagulant activity); ↓ ERK/Egr-1- and JNK/AP-1-mediated transactivation of TF promoter activity			[37]
Wogonin and wogonoside		In vitro and in vivo (ICR mice)	↑ APTT and PT prolongation (anticoagulant activity); ↓ fibrin polymerization; ↓ mouse platelet aggregation induced by thrombin; ↓ amidolytic activity of thrombin; ↓ FXa; ↓ thrombin production from prothrombin; ↑ prolongation of tail bleeding time			[58]
GTC, EGCG		In vitro*, *in vivo (male ICR mice), ex vivo (male Sprague–Dawley rats)	Protection of paralysis and death due to pulmonary thrombosis; prolonged mouse tail bleeding time; ↓ platelet aggregation			[59]
Epicatechin		Healthy volunteers’ plasma samples (*n* = 10)	↓ Maximal platelet aggregation; ↓ ETP; ↑ fibrinolysis;			[15]
Dark chocolate (flavan-3-ols)		Healthy volunteers (*n* = 18) ingested 50 g of 90% cocoa chocolate within 5 min	↑ Collagen/ADP-induced closure time (and correlation with the increase of total SREMs); suggested a beneficial protective role for subjects at increased risk of thrombosis			[60]
Flavonoid-rich dark chocolate		Healthy men receiving placebo (*n* = 34) or 50 g of flavonoid-rich dark chocolate group (*n* = 31)	↓ Acute prothrombotic response to psychosocial stress (↓ stress reactivity of D-dimer)			[61]
Isoquercetin		Advanced cancer patients at high risk for thrombosis, receiving isoquercetin at 500 mg (*n* = 28) or 1000 mg (*n* = 29)	↓ D-dimer; ↑ PDI inhibitory activity; ↓ platelet-dependent thrombin generation			[63]
**Phenolic acids**						
Caffeic acid		Human platelets	↓ Collagen-induced platelet aggregation; ↓ Ca^2+^ mobilization; ↓ adenosine 1,4,5-tri-phosphate release; ↓ expression of P-selectin; ↓ αIIbβ3 activation; ↓ phosphorylation of AKT and ERK; ↑ cAMP level			[72]
		Washed platelets from rats (Sprague–Dawley)	↓ Collagen-induced platelet aggregation; ↓ Ca^2+^ and TXA_2_ levels; ↑ cAMP level			[73]
Curcumin		Healthy volunteers plasma samples; HUVEC cell lines	↑ APTT and PT; ↓ thrombin and FXa generation			[75]
		Male ICR mice (6 weeks) and washed human platelets	↓ Thrombin-stimulated platelet activation; ↓ αIIbβ3/FIB and Akt phosphorylation			[77]
p-Hydroxy-cinnamic acid		Healthy volunteers plasma samples	↑ APTT			[76]
Ferulic acid		Male ICR mice (6 weeks) and washed platelets from mice	↓ Risk of death caused by pulmonary thrombosis; ↓ platelet aggregation; ↓ Ca2^+^ mobilization; ↓ TXB2 generation; ↑ levels of cAMP and cGMP; ↑ phosphorylation of vasodilator-stimulated phosphoprotein (VASP), ↓ phosphorylation of MAPK and PDE			[78]
		Venous blood of Japanese White Rabbits	↑ Whole blood coagulation time			[80]
5-(Hydroxymethyl)furfural		Whole blood samples were collected from individuals with sickle cell trait	↓ The effects of hypoxia, ↓ risk for venous thromboembolism, ↓ blood rheology, restoration of near-normal flow velocities at very low oxygen			[83]
Dan Zhi tablet		New Zealand white rabbits, ICR mice, Sprague–Dawley rats as a model of middle cerebral artery occlusion	↑ Antiplatelet activity, ↓ prostaglandin G/H synthase 1 activity, ↓ platelet aggregation in vitro, ↓ ADP- or AA-induced ex vivo platelet aggregation, ↓ thromboxane A2, ↑ prevention against thrombus formation			[84]
**Stilbenes**						
Resveratrol	Male Sprague–Dawley rats			↑ Anticoagulant effect of warfarin; ↓ BCRP and CYP2C9	[88]	
	Male C57BL/6 J			↑ Anticoagulant effect of warfarin	[89]	
	HUVEC cell line			↓ IL-8, t-PA-1, and von Willebrand factor expression and secretion; ↓ factor VIII activity	[90]	
	Male Sprague–Dawley rats			↓ Incidence of PVST after splenectomy; ↓ platelet aggregation and ROS production; ↑ NO generation and platelet apoptosis	[91]	
**Coumarins**						
Esculetin	Human platelets and male ICR mice			↑ Prevention of thrombosis, ↓ PLCγ2-PKC-AKT activation, ↓ collagen- and arachidonic acid-induced platelet aggregation, ↓ ATP release, ↓ P-selectin expression, ↓ hydroxyl radical formation, ↓ mortality, ↑ occlusion time in thrombotic platelet plug formation	[96]	
3-(5-Hydroxy-2,2-dimethyl-chroman-6-yl)-N-{2-[3-(5-hydroxy-2,2-dimethyl-chroman-6-yl)-propionylamino]-ethyl}-propionamide	Male Golden Syrian hamster (100–120 g), Male Sprague–Dawley rats (160–180 g), and male Swiss mice (18– 20 g)			↑ Prevention of collagen- and epinephrine-induced pulmonary thromboembolism, ↑ prevention of arachidonic acid-induced death, ↓ thrombus weight, ↓ ADP and collagen-induced platelet aggregation, ↓ whole-blood aggregation	[97]	
**Alkaloids**						
Rutaecarpine		Male ICR mice (6 weeks) and washed human platelets	↑ Antiplatelet activation, ↓ PLCγ2/PKC, PI3K/Akt/GSK3β, ↓ P-selectin expression, ↓ ATP release, ↓ [Ca^2+^] immobilization, ↓ hydroxyl radical formation			[100]
Coffee (methylxanthines)		Patients with a first venous thrombosis (*n* = 1803) and controls (*n* = 1803)	↓ Risk of venous thrombosis, ↓ von Willebrand factor, ↓ factor (F) VIII			[101]
Rhizoma of Corydalis yanhusuo		Rabbit platelets	↓ Thrombin-induced platelet aggregation			[103]
**Saponins**						
D39		HUVEC cells and inferior vena cava ligation injury in mice	↓ Procoagulant activities, ↓ tissue factor expression, ↓ thrombus weight, ↓ deep vein thrombosis, ↓ NMMHC IIA, ↓ NF-κB, ↑ Akt/GSK3β			[105]
Diosgenyl b-D-galactopyranosyl-(1 → 4)-b-D-glucopyranoside		Male Balb/C mice (four weeks old, 18–22 g) and male Wistar rats (eight weeks old, 200–250 g)	↓ Platelet aggregation, ↓ factor VIII activities, ↑ APTT, ↑ protection rate, ↑ antithrombotic activity			[106]
Panaxatriol saponin and ginsenosides (Rg1, Re, and R1)		The human blood or rabbit (New Zealand albino rabbits) blood–platelet preparation	↑ Antiplatelet activity, ↓ platelet aggregation, ↓ intracellular calcium mobilization, ↓ ERK2, and p38 phosphorylation			[107]
**Iridoid glycosides**						
Gardenia jasminoides		Rat model	↑ Prolonged bleeding time, ↓ platelet aggregation, ↓ thrombosis			[110]
Geniposide and genipin		Male ICR mice	↑ Antithrombotic activity, ↓ collagen-induced platelets aggregation, ↑ the time required for thrombotic occlusion, ↓ phospholipase A(2)			[111]
Zhizi (Gardeniae fructus)		Rat model of carotid artery thrombosis	↑ Antithrombotic action, ↓ collagen-induced platelet aggregation, ↓ arterial thrombus, ↑ thrombin time			[112]
**Sesquiterpenes**						
Sesquiterpene glycoside and ferulic acid from the leaves of *Eriobotrya japonica* Lindley (Rosaceae)		Sprague–Dawley rats, a microsomal fraction of rat lung tissue as the tissue factor source	↓ Tissue factor activity, ↑ prothrombin time in the presence of tissue factors in a dose-dependent manner			[114]
Curdione		Human platelets	↓ Human platelet aggregation, ↓ phosphorylated AMPK, ↓ P-integrin, ↓ vinculin/talin-mediated integrin αIIbβ3 signaling pathway			[115]
Nootkatone		Platelet aggregation in whole blood (in vitro) and pial arterioles and venules (in vivo)	↓ Prothrombotic effect, ↓ plasma concentration of fibrinogen, ↓ plasminogen activator inhibitor-1, ↓ IL-6, ↓ lipid peroxidation, ↑ activated partial thromboplastin time, ↑ prothrombin time, ↓ thrombogenicity, ↓ systemic and cardiac oxidative stress and DNA damage, ↑ nuclear factor erythroid-derived 2-like 2, ↑ heme oxygenase-1			[116]
**Sulfated polysaccharides**						
Sulfated galactans (*Acanthophora muscoides*)		Animal models of experimental thrombosis	Serpin-independent anticoagulant activities, FXII-related procoagulant effects, ↓ arterial thrombus formation, ↑ venous thrombosis			[118]
Sulfated D-galactans (Botryocladia occidentalis)		Platelet-depleted animals	Lower dose: anticoagulant activity, prevent thrombosis, ↑ thrombin, and factor Xa inhibition Higher dose (more than 0.5 mg/ kg body weight): ↑ platelet aggregation			[119]
Sulfated pyranosic (1- > 3)-β-L-arabinan (*Codium vermilara*)		Samples containing 1 μm human thrombin	↑ anticoagulant activity, direct interaction with thrombin			[120]
**Plant foods**						
*Ginkgo biloba* constituents		Fluorescence-based biochemical assay (screening and characterization of each constituent); inhibition kinetics (inhibitory effects on human thrombin); molecular docking (interaction between biflavones and thrombin); mass spectrometry-based lysine labeling reactivity assay (identification of ligand-binding sites)	Thrombin-inhibitory activity of Ginkgo biflavones (ginkgetin, isoginkgetin, bilobetin, and amentoflavone) and flavonoids (luteolin, apigenin, quercetin, kaempferol, and isorhamnetin); Ginkgo biflavones → occupation of the active cavity with strong interactions of salt bridges and hydrogen bonds and binding on human thrombin at exosite I rather than exosite II			[122]
GBE50 combined with aspirin		In vitro	↑ Antiplatelet effects (synergistic and additive effects in restraining platelet aggregation			[123]
Ginkgo biloba extract		HUVECs cell model	↑ Antithrombotic effects (increased thrombomodulin expression and tissue-type plasminogen activator secretion); KLF2 suggested a key factor of these mechanisms			[124]
Chamomilla aqueous extract		Blood from healthy volunteers	↑ Antiplatelet effects (inhibition of ADP-induced and collagen-induced platelet aggregation)			[126]
Polysaccharide-polyphenolic conjugates isolated from MC		Platelet-rich plasma from healthy donors and donors with cardiovascular disorders; in vitro (cytotoxicity)	↓ Platelet aggregation (healthy donors); ↓ platelet aggregation (donors with cardiovascular disorders); no cytotoxicity effects on human blood platelets, mouse fibroblast cultures L929 and human lung cells A549			[127]
A. ursinum and A. sativum extracts		Blood from healthy volunteers	↓ Platelet aggregation via inhibition ADP pathway			[135]
A. atroviolaceum extract		Blood from healthy volunteers	↓ Platelet aggregation after induction by platelet aggregation inducers (arachidonic acid and ADP)			[136]
Garlic tablets		Randomized controlled clinical trial (*n* = 62 healthy volunteers). Platelet aggregation was evaluated by light transmittance aggregometry	No association between the consumption of garlic tablets and platelet aggregation			[137]
Flavonolignans isolated from Silybum marianum L		Blood from healthy volunteers	↓ Platelet aggregation, ↓ P-selection expression, ↓ activation of αIIbβ3			[140]
Flavonolignans isolated from Silybum marianum L		Blood from healthy volunteers	↓ Platelet aggregation, ↓ COX activity, ↓ malondialdehyde, and thromboxane A_2_ levels			[141]
Silybum marianum L flavonolignans and their sulfated conjugates		Blood from healthy volunteers; isolated rat aortas	↑ Vasorelaxant effect ex vivo in rat aortas; ↓ platelet activity			[142]

*Explanatory notes:* ↓, inhibition, suppression, reduction; ↑, promotion, enhancement; → leading to, resulting in

*8-OHdG*, 8-hydroxy-2'-deoxyguanosine; *AA*, arachidonic acid; *ADP*, adenosine diphosphate; *AKT*, protein kinase B; *APTT*, activated partial thromboplastin time; *ATP*, adenosine triphosphate; BCRP, breast cancer resistance protein; *Ca*^*2*+^, calcium; *cAMP*, cyclic adenosine monophosphate; *cGMP*, cyclic guanosine monophosphate; *CYP2C9*, cytochrome P450 family 2 subfamily C member 9; *ECs*, endothelial cells; *EGCG*, (-)-epigallocatechin gallate; *ERK2*, extracellular signal-regulated kinase 2; *ETP*, endogenous thrombin potential; *FXa*, activated factor X; *GBE50*, Ginkgo biloba extract 50; *GPCR*, G-protein coupled receptor; *GPVI*, glycoprotein VI; *GSK3β*, glycogen synthase kinase-3 beta; *GTC*, green tea catechins; *HUVECs*, human umbilical vein endothelial cells; *IL-8*, interleukin 8; *KLF2*, Krüppel-like factor 2; *LAT*, linker for activation of T cells; *MC*, *Matricaria chamomilla* L.; *NF-κB*, nuclear factor kappa-light-chain-enhancer of activated B cells; *NMMHC IIA*, non-muscular myosin heavy chain IIA; *NO*, nitric oxide; *p38*, p38 mitogen-activated protein kinase; *PDI*, protein disulfide isomerase; *PI3K*, phosphoinositide 3-kinase; *PKC*, protein kinase C; *PLCγ2*, phosphatidylinositol-specific phospholipase Cγ2; *PT*, prothrombin time; *PVST*, portal vein system thrombosis; *Rutin@AgNPs*, rutin-loaded silver nanoparticles; *SREMs*, structurally related (epi)catechin metabolite; *Syk*, spleen tyrosine kinase; *TNF-α*, tumor necrosis factor-α; *t-PA-1*, tissue plasminogen activator-1; *TXA2*, thromboxane A2; *TXB2*, thromboxane B2; *VASP*, vasodilator-stimulated phosphoprotein

In conclusion, phytochemicals show potent antithrombotic efficacy demonstrated in numerous in vitro, ex vivo, and in vivo models as well as in clinical evaluations conducted on healthy individuals and persons at high risk of thrombotic events, such as cancer patients.

## COVID-19 and the risk of thrombotic events: the potential role of phytochemicals

The vascular system is, in particular, susceptible to SARS-CoV-2 infections [143] owing to the presence of endothelial cell (ECs) surface ACE2 receptors through which the SARS-CoV-2 virus gains access to the ECs [144–148]. Impairment of vascular integrity and function owing to the intracellular presence of SARS-CoV-2 particles and host inflammatory cells in the ECs is suggestive that the alterations in the endothelium function (endothelial dysfunction; ED) contributes to disease progression and outcome among COVID-19 patients [144, 146–148]. In addition, apart from the ECs, SARS-CoV-2 was found to infect the pericytes and cause a leaky endothelial barrier [149].

The endothelial dysfunction should explain why individuals with COVID-19 are at high risk for venous and arterial thrombotic events [150], particularly the patients with a severe course of disease [151]. The risk is visible when comparing critically ill patients with COVID-19 and non-COVID-19 respiratory diseases [152]. SARS-CoV-2 infection triggers a process known as immunothrombosis. Critically ill COVID-19 ICU patients reportedly present with venous thromboembolism and microvascular lung thrombosis. These conditions could be correlated to the elevated levels of D-dimer, von Willebrand factor (VWF), fibrinogen, and soluble P-selectin and increased VWF and factor VIII activity when compared with their non-ICU counterparts [143, 147, 148, 153–157]. This, in turn, can also explain the characteristic ED, hyper-viscosity/coagulation, increased rates of thrombotic events, and microvascular complications such as venous thromboembolism, microvascular lung thrombosis, arterial events, and disseminated intravascular damage which required ICU admission and critical care and higher mortality among severe COVID-19 patients [143, 146–148, 153, 154, 156–159].

In particular, ED in COVID-19 patients becomes more relevant in diabetic subjects with diabetes-associated ED correlated to several diabetes-related vascular complications. Diabetes-linked hyperinsulinemia, hyperglycemia, and increased levels of free fatty acids can contribute to significant metabolic/molecular changes in ECs, causing ED [147, 148, 160]. It is well known that diabetic COVID-19 patients have a more severe course of the disease, require ICU admissions and critical care, and have higher mortality when compared to non-diabetic COVID-19 patients [147, 148, 155]. It is difficult to delineate whether COVID-19 infection accentuates diabetes-associated ED or diabetes-associated ED exacerbates COVID-19 infection and needs further investigation.

Innate and adaptive immunity regulates the pathophysiology of SARS-CoV-2-induced endothelial and platelet dysfunctions through numerous direct or indirect mechanisms. In this regard, SARS-CoV-2 modulates numerous immune-modulatory cytokines and chemokines that trigger the coagulation processes and create an intravascular prothrombotic environment that is manifested with pulmonary embolism and thrombocytopenia. During this process, stimulated monocytes and neutrophils interact with platelets and affect the coagulation cascade, leading to clot formation in small and larger blood vessels [161]. Consequently, microthrombotic intravascular events may contribute to acute respiratory distress syndrome (ARDS) and dysfunctions in other organs. The direct SARS-CoV-2-induced endothelial injury with a dysregulated coagulation factor stimulation and hyper-activated immune system is postulated as crucial contributors to the development of the COVID-19-linked prothrombotic intravascular conditions [162].

Therefore, therapeutic intervention(s) such as anticoagulant therapy and antioxidant therapy that restores and stabilizes the normal endothelial function and target inflammation may improve the prognosis and survival of COVID-19 patients [163]. In this regard, several phytochemicals known to possess anti-inflammatory, antioxidant, and anti-coagulative properties could mend the impaired EC function in COVID-19 patients.

Thromboprophylaxis in COVID-19 patients is a highly discussed topic among clinicians. The recommendations are derived from limited clinical evidence from observational studies [164]. Anticoagulation therapy in COVID-19 patients with a severe course of disease using therapeutic doses of heparin might lower the thrombotic complications without increasing the risk of bleeding [165]. However, the therapy with heparin is significantly linked with some adverse effects in an organism, such as thrombocytopenia, hypersensitivity reactions hypoaldosteronism, or osteoporosis [166]. Therefore, from the clinical point of view, it is essential to set up specific therapeutic schemes, including optimal dosage for effective and safe anticoagulant/antithrombotic drugs in the prevention and treatment of coagulation events associated with COVID-19 disease. As mentioned above, new alternative anticoagulant/antithrombic molecules include phytochemicals demonstrating various cell biological activities. In addition, the plant-derived compounds were previously documented to have anti-viral activities against the SARS family. The cardioprotective activities of phytochemicals include the inhibition of platelet activation, secretion, adhesion, and aggregation, thus reducing the processes of thrombus formation and consequent vascular occlusion [167]. Based on mentioned mechanisms, specific phytochemicals can target the platelet intracellular Ca^2+^ mobilization, thromboxane synthesis, and phospholipases-mediated MAPK signaling that induces the suppression of platelet functions [167]. Based on these well-described mechanisms, phytochemicals could be potentially beneficial in the treatment of COVID-19 patients, even though selected phytochemicals represent excellent candidate molecules for anticoagulant therapy within the primary and secondary prevention of thromboembolic events, as the first well-designed clinical studies must be performed to apply them into clinical practice.

## Conclusions and expert recommendations in the framework of 3P medicine

Currently applied antithrombotic drugs used, e.g., to protect affected individuals against ischemic stroke demonstrate significant limitations. For example, platelet inhibitors possess only moderate efficacy. On the other hand, thrombolytics and anticoagulants significantly increase hemorrhage [168]. Contextually, new approaches are currently under consideration to develop next-generation antithrombotics with improved efficacy and more personalized and targeted application. Below clinically relevant conditions are exemplified following PPPM principles.

### Exemplified clinically relevant conditions: primary, secondary, and tertiary care

Epidemiologic analysis of cerebral venous sinus thrombosis (CVST) in the USA updated for the years 2018–2019 revealed a higher prevalence of ischemic and hemorrhage strokes and other types of venous thromboembolism, infections of central nervous and head/neck systems, thrombophilia (both genetic and acquired), cancers, head injury, and hemorrhagic and connective tissue disorders as strongly predisposing to CVST [169]. Furthermore, CVST has been observed more commonly in a young stroke (young adults and children) compared to arterial stokes. Adult females aged below 49 years and diagnosed with CVST demonstrate significantly higher pregnancy prevalence compared to the age-adjusted general population [169]. Contextually, the accumulated knowledge exemplified with the CVST well justifies the practical application of corresponding health risk assessment tools to predict thrombosis-related health risks, followed by targeted protection tailored to the individualized patient profiling.

### Primary care

Pregnancy and puerperium are reported as increasing the risk of ischemic and hemorrhagic stroke demonstrating a three-fold higher incidence compared to non-pregnant women. To this end, the need for new strategies to improve predictive approach and personalized protection is well justified in recent articles [170, 171]. Maternal CVST and ischemic stroke defined as occurring during pregnancy or in postpartum are the major cause of maternal disability and mortality [171]. Per evidence, pregnancy-related physiologic changes increase the risk of thromboembolic events including CVST. In practical medicine, there is an evident lack of predictive services which would help to stratify affected women at high risk for pregnancy-related stroke who require pre-pregnancy check-up, closer monitoring, and effective individualized protection during pregnancy and postpartum [172].

During pregnancy, the maternal cardiovascular system is exposed to hemodynamic stress which is a significant risk factor influencing the health status of the mother and offspring [173, 174]. To this end, sub-optimal maternal health conditions overlooked in pre-pregnancy time may lead to progressive abnormalities in the fetal development and maternal health status during pregnancy and postpartum. Recently published study “*Pre-pregnancy check-up of maternal vascular status and associated phenotype is crucial for the health of mother and offspring*” investigated maternal pre-pregnancy low BMI and Flammer syndrome phenotype which were co-diagnosed with the connective tissue dysfunction, increased stiffness of peripheral vessels and concomitant systemic effects increasing risks of ischemic stroke at a young age, among others [175]. Contextually, Flammer syndrome phenotyping has been strongly recommended for PPPM-relevant pre-pregnancy check-up as well as an application of natural substances in primary and secondary care to mitigate associated health risks of the affected mother and offspring [175]. The detailed analysis demonstrated positive effects of phytochemicals protecting against oxidative stress, endothelial dysfunction, connective tissue dysfunction, small vessels disease and pro-inflammatory conditions – all involved in the pathomechanisms underlying CVST and ischemic stroke [175, 176].

### Secondary and tertiary care

#### Cancer-associated thrombosis

Thromboembolism significantly impacts individual outcomes in patients with malignancies. On the one hand, cancer pathomechanisms are intimately related to thrombosis resulting from the complex interplay between coagulation abnormalities, activated adhesion and platelets, endothelial dysfunction, and activated pro-inflammatory pathways. On the other hand, vascular toxicity and dysfunction are frequent adverse effects of the currently applied anti-cancer therapy. New predictive and therapeutic strategies are strongly recommended for comprehensive secondary care considering the predictive approach, an aggressive antithrombotic treatment, and cost-effective protection tailored to the personalized patient profile [177, 178]. To this end, phytochemicals have been demonstrated as being safe and potentially highly effective in protecting against non-physiologic inflammation, endothelial dysfunction, and cancer-associated stroke [19, 179–181].

### Thrombosis in COVID-19-infected individuals

Acute cerebrovascular disease is not a rare complication in COVID-19-affected individuals, particularly with pre-existing vascular risk factors. Cerebral thrombosis and thromboembolism have been suggested as possible disease causality with corresponding pathomechanisms of hypercoagulable conditions and infection-induced systemic inflammatory response synergistically increasing thrombosis and stroke risks [182]. Post-mortem analysis of patients who died on COVID-19 multi-organ dysfunction provided evidence of endothelial disruption, extensive mononuclear and neutrophil infiltration into large arteries, and excessive platelet activation - all relevant for thrombosis. Recent studies emphasize the relevance of corresponding pathomechanisms also for young adults [183]. Phytochemicals are considered as potentially highly effective against non-physiologic inflammation and endothelial dysfunction in the protection of stratified individuals predisposed to the severe disease cause [184].

## Data Availability

Not applicable.

## Abbreviations

3P: Predictive preventive personalized
8-OHdG: 8-Hydroxy-2'-deoxyguanosine
AA: Arachidonic acid
AC: Adenylyl cyclase
ADP: Adenosine diphosphate
AKT: Protein kinase B
AP-1: Activator protein-1
APTT: Activated partial thromboplastin time
ARDS: Acute respiratory distress syndrome
ATP: Adenosine triphosphate
BCRP: Breast cancer resistance protein
BDMC: Bisdemethoxycurcumin
BMI: Body mass index
Ca^2+^: Calcium
cAMP: Cyclic adenosine monophosphate
cGMP: Cyclic guanosine monophosphate
COVID-19: Coronavrus disease 2019
COX1/2: Cyclooxygenase 1/2
CVST: Cerebral venous sinus thrombosis
CYP2C9: Cytochrome P450 family 2 subfamily C member 9
DOACs: Direct oral anticoagulants
DVT: Deep vein thrombosis
ECs: Endothelial cells
ED: Endothelial dysfunction
EGCG: (-)-Epigallocatechin gallate
Egr-1: Early growth response-1
eNOS: Endothelial NOS
ERK2: Extracellular signal-regulated kinase 2
ETP: Endogenous thrombin potential
FXa: Activated factor X
GBE50: Ginkgo biloba Extract 50
G-hesperidin: Glucosyl hesperidin
GPCR: G-protein coupled receptor
GPVI: Glycoprotein VI
GSK: Glycogen synthase kinase
GSK3β: Glycogen synthase kinase-3 beta
GTC: Green tea catechins
HUVECs: Human umbilical vein endothelial cells
ICU: Intensive care unit
IL: Interleukin
IP: Prostacyclin receptor
IP_3_R: Inositol 1,4,5-trisphosphate
JAK: Janus kinase
JNK: C-Jun N-terminal kinase
KLF2: Krüppel-like factor 2
LAT: Linker for activation of T cells
MAPKs: Mitogen-activated protein kinases
MC: *Matricaria chamomilla* L.
MeSH: Medical subject heading
MI: Myocardial infarction
mTOR: Mammalian target of rapamycin
NFκB: Nuclear factor kappa-enhancer of activated B cells
NMMHC IIA: Non-muscular myosin heavy chain IIA
NO: Nitric oxide
p38: Mitogen-activated protein kinase
P-AMPK: Phosphorylated AMPK
PAR: Protease-activated receptors
PDEs: Phosphodiesterase
PDI: Protein disulfide isomerase
PE: Pulmonary embolism
PGG2: Prostaglandin G2
PGH2: Prostaglandin H2
PGI2: Prostaglandin I2
PI3K: Phosphoinositide 3-kinase
PKA: CAMP-dependent protein kinase
PKB: Protein kinase B
PKC: Protein kinase C
PKG: CGMP-dependent protein kinase
PLA(2): Phospholipase A(2)
PLCγ2: Phosphatidylinositol-specific phospholipase Cγ2
PPARs: Peroxisome proliferator-activated receptors
PPPM: Predictive preventive personalized medicine
PT: Prothrombin time
PVST: Portal vein system thrombosis
ROS: Reactive oxygen species
Rutin@AgNPs: Rutin-loaded silver nanoparticles
SFK: Src family kinase
sGC: Soluble guanylyl cyclase
SREMs: Structurally related (epi)catechin metabolite
Syk: Spleen tyrosine kinase
TF: Tissue factor
TNF-α: Tumor necrosis factor-α
TP: Thromboxane receptor
t-PA-1: Tissue plasminogen activator-1
TXA2: Thromboxane A2
TXB2: Thromboxane B2
TX-synthase: Thromboxane synthase
VASP: Vasodilator-stimulated phosphoprotein
VSMCs: Vascular smooth muscle cells
VWF: Von Willebrand factor
WBCT: Whole blood coagulation time
